# Bone‐marrow‐derived mesenchymal stem cells inhibit gastric aspiration lung injury and inflammation in rats

**DOI:** 10.1111/jcmm.12866

**Published:** 2016-04-07

**Authors:** Jing Zhou, Liyan Jiang, Xuan Long, Cuiping Fu, Xiangdong Wang, Xiaodan Wu, Zilong Liu, Fen Zhu, Jindong Shi, Shanqun Li

**Affiliations:** ^1^ Department of Respiratory MedicineZhongshan HospitalFudan UniversityShanghaiChina; ^2^ Department of General PracticeZhongshan HospitalFudan UniversityShanghaiChina; ^3^ Department of Respiratory MedicineShanghai Chest HospitalShanghai Jiaotong UniversityShanghaiChina; ^4^ Department of Respiratory MedicineThe Fifth People's Hospital of ShanghaiFudan UniversityShanghaiChina

**Keywords:** bone marrow mesenchymal stem cell, gastric aspiration, acute respiratory distress syndrome, 15‐deoxy‐Δ12, 14 prostaglandin J_2_, peroxisome proliferator‐activated receptor‐γ

## Abstract

Gastric aspiration lung injury is one of the most common clinical events. This study investigated the effects of bone‐marrow‐derived mesenchymal stem cells (BMSCs) on combined acid plus small non‐acidified particle (CASP)‐induced aspiration lung injury. Enhanced green fluorescent protein (EGFP ^+^) or EGFP ^−^ BMSCs or 15d‐PGJ_2_ were injected *via* the tail vein into rats immediately after CASP‐induced aspiration lung injury. Pathological changes in lung tissues, blood gas analysis, the wet/dry weight ratio (W/D) of the lung, levels of total proteins and number of total cells and neutrophils in bronchoalveolar lavage fluid (BALF) were determined. The cytokine levels were measured using ELISA. Protein expression was determined by Western blot. Bone‐marrow‐derived mesenchymal stem cells treatment significantly reduced alveolar oedema, exudation and lung inflammation; increased the arterial partial pressure of oxygen; and decreased the W/D of the lung, the levels of total proteins and the number of total cells and neutrophils in BALF in the rats with CASP‐induced lung injury. Bone‐marrow‐derived mesenchymal stem cells treatment decreased the levels of tumour necrosis factor‐α and Cytokine‐induced neutrophil chemoattractant (CINC)‐1 and the expression of p‐p65 and increased the levels of interleukin‐10 and 15d‐PGJ_2_ and the expression of peroxisome proliferator‐activated receptor (PPAR)‐γ in the lung tissue in CASP‐induced rats. Tumour necrosis factor‐α stimulated BMSCs to secrete 15d‐PGJ_2_. A tracking experiment showed that EGFP ^+^ BMSCs were able to migrate to local lung tissues. Treatment with 15d‐PGJ_2_ also significantly inhibited CASP‐induced lung inflammation and the production of pro‐inflammatory cytokines. Our results show that BMSCs can protect lung tissues from gastric aspiration injury and inhibit lung inflammation in rats. A beneficial effect might be achieved through BMSC‐derived 15d‐PGJ_2_ activation of the PPAR‐γ receptor, reducing the production of proinflammatory cytokines.

## Introduction

Gastric aspiration is a common clinical event. The content of the aspirate includes gastric contents, blood, bacteria, liquids and secretions. Gastric aspiration is a major direct cause of acute lung injury (ALI) and the more severe acute respiratory distress syndrome 1, 2. Gastric aspiration may result in a variety of clinical lung injuries, ranging from mild, subclinical pneumonitis to a more severe, progressive disease with associated high mortality. Acute respiratory distress syndrome from gastric aspiration frequently occurs in an unconscious patient in one of every 2000–4000 anaesthetic cases 3, 4. Acute respiratory distress syndrome associated with gastric aspiration accounts for approximately 30% of the mortality and 20% of all deaths attributable to anaesthesia 5.

Acid and food particles are the main components of gastric aspiration that induces lung injury. Food particles of gastric aspirates are not only responsible for airway obstruction but also directly contribute to inflammatory damage 6. The aspiration of combined acid and small gastric particles (CASP) can lead to more severe lung injury in contrast to lung injury induced by either acid or gastric particles alone and can generate more severe lung pathological changes than the sum of the individual components 4, 6. Inflammatory injury is an important characteristic in gastric aspiration lung injury. Acid‐induced lung injury is characterized by the stimulation of capsaicin‐sensitive neurons and a direct chemical acid burn on the airway epithelium followed by an acute neutrophilic inflammatory response over 4–6 hrs 1. Therefore, reducing or blocking the inflammatory cascade in gastric aspiration lung injury is an important treatment strategy 7.

15‐Deoxy‐Δ‐12,14‐prostaglandin J_2_ (15d‐PGJ_2_) has anti‐inflammatory effects 8. 15d‐PGJ_2_ is formed by spontaneous PGD_2_ dehydration into cyclopentane (PGJ_2_ is forms first, then further dehydration leads to Δ‐12‐PGJ_2_ and finally to 15d‐PGJ_2_). In contrast to other prostaglandins, 15d‐PGJ_2_ may exert anti‐inflammatory effects mainly through interacting with intracellular targets 9. In particular, 15d‐PGJ_2_ is an endogenous ligand for the intranuclear receptor PPARγ. PPARγ is a member of the nuclear receptor superfamily and a ligand‐activated transcription factor, with pleiotropic effects on lipid metabolism, inflammation and cell proliferation. PPARγ is expressed in both alveolar macrophages and neutrophils and plays a critical role in inflammatory responses. Many studies have demonstrated that the activation of PPARγ by 15d‐PGJ_2_ or other agonists has anti‐inflammatory effects and leads to a substantial reduction in experimental ALI 9, 10, 11. However, the roles of 15d‐PGJ_2_ and PPARγ in gastric aspiration‐induced lung injury are unknown.

Recently, bone‐marrow‐derived mesenchymal stem cells (BMSCs) have attracted a great deal of attention because they have a potent *in vivo* immunosuppressive effect in humans, as reported in 2004 12. Bone‐marrow‐derived mesenchymal stem cells exhibit anti‐inflammatory effects in several diseases, including myocardial infarction, diabetes, sepsis, hepatic failure and acute renal failure. The compelling benefits of BMSCs for lung injury have also generated a great deal of interest 13. Bone‐marrow‐derived mesenchymal stem cells can increase the release of prostaglandin E_2_, which acts on the EP_2_ and EP_4_ receptors of macrophages and stimulates the production and release of interleukin (IL)‐10 14. However, the effects and underlying mechanisms by which BMSCs exert anti‐inflammatory effects on CASP‐induced aspiration lung injury are unknown. In this study, we investigated the effects of BMSCs on CASP‐induced aspiration lung injury in a rat model.

## Materials and methods

### BMSC isolation and culture

Bone‐marrow‐derived mesenchymal stem cells were isolated from rat bone marrow. Briefly, three specific‐pathogen‐free (SPF) male Sprague–Dawley rats weighing 100–150 g were anaesthetized by an intraperitoneal injection of 10% chloral hydrate (4 ml/kg) and then executed by breaking the neck; the bone marrow tissue was harvested from bilateral femurs under sterile conditions. Cells were plated in 25 cm^2^ plastic flasks with DMEM‐LG (Gibco, New York, NY, USA) containing 10% foetal bovine serum (FBS; Gibco), 10 ng/ml epidermal growth factor (Sigma‐Aldrich, Saint Louis, MO, USA), 100 U/ml penicillin (Gibco) and 100 U/ml streptomycin (Gibco) at 37°C in humid air with 5% CO_2_ (Precision Scientific, MA, USA) for culture. After a 48‐hr incubation, BMSCs adhered to the bottom of the culture plates and haematopoietic cells remained suspended in the medium; non‐adherent cells were discarded. The medium was changed every 3–4 days thereafter. When the adherent cells were confluent to more than 80%, they were digested with 0.25% trypsin‐EDTA (Gibco) and re‐plated at 1:2 dilutions under the same culture conditions. The cells were further purified by passaging. The growth curves of BMSCs at passages 2, 4 and 6 were determined by a CCK‐8 kit (Dojindo Chemical Co., Kyushu, Japan) in accordance with the manufacturer's instructions.

### Immunophenotypic analysis of BMSCs

The BMSCs at passage 5 were trypsinized into a single cell suspension and re‐cultured in multi‐well tissue culture plates (4 wells) containing Poly‐l‐Lysine (Sigma‐Aldrich, Munich, Germany)‐pre‐coated cover slides at a density of 1 × 10^3^ cells/well in DMEM medium supplemented with 10% FBS. The cultures were maintained in a CO_2_ incubator under the same culture conditions until they reached confluence. Cell BMSCs were harvested by digestion with trypsin, washed with PBS, and stained with PE‐ or fluorescein isothiocyanate (FITC)‐labelled antibodies to CD14, CD19, CD34, CD45, CD73, CD90 or CD105 (BioLegend, Shanghai, China). The BMSCs at passages 5 were used for flow cytometry (Beckman Coulter, Brea, CA, USA) using Expo32 ADC analysis software, concurrent with the time that they were used during the whole experiment. Bone‐marrow‐derived mesenchymal stem cells were positive for CD73, CD93 and CD105 and negative for the haematopoietic markers CD14, CD19, CD34 and CD45 15, 16.

### Animal models

Specific‐pathogen‐free male Sprague–Dawley rats weighing 200–250 g (Shanghai Slac laboratory animal Co., Ltd., Shanghai, China) were divided into three groups: (*i*) Normal saline (NS) + PBS group: 12 rats were injected with PBS *via* the tail vein immediately after being instilled with NS intratracheally; (*ii*) Injury + PBS group: 12 rats were injected *via* the tail vein with PBS immediately after being instilled with CASP intratracheally; and (*iii*) Injury + BMSC group: 12 rats were injected *via* the tail vein with BMSCs immediately after being instilled with CASP intratracheally. All the animals were maintained at the Experimental Animal Center of Zhongshan Hospital, Fudan University. All the animal protocols were reviewed and approved by the ethical committee for Animal Care and Use at Zhongshan Hospital, Fudan University. All the animals were housed in high‐efficiency particulate air‐filter‐topped cages in a sterile laminar flow environment and had free access to food and water before and after the operation.

### CASP‐induced aspiration lung injury and BMSC transplantation

Gastric particulates were prepared from the stomach contents of SPF male Sprague–Dawley rats as described by Knight 17. Briefly, five SPF male Sprague–Dawley rats weighing 100–150 g were anaesthetized by an intraperitoneal injection of 10% chloral hydrate (4 ml/kg) and were then killed by breaking the neck; the stomach contents were collected, washed several times in NS, filtered through gauze and sterilized by autoclaving (final particle sizes were <30 μm). Then, CASP was made with hydrochloric acid (pH = 1.25, 20 mg/ml). The recipient rats were anaesthetized by the intraperitoneal injection of 10% chloral hydrate (4 ml/kg). After anaesthesia, the trachea of each rat was exposed with a 1.00 cm longitudinal incision, and a 16 gauge needle was introduced through the tracheal cartilage. A catheter was then advanced through the needle, and the prepared combination suspension of CASP (1.5 ml/kg) or an equal volume of NS was injected intratracheally. The BMSCs (5 × 10^6^) at passages 5 were suspended in 0.5 ml of PBS and injected *via* the tail vein of rats immediately after the induction of CASP‐induced aspiration lung injury. The animals were monitored after BMSC transplantation.

### EGFP‐BMSC trafficking experiment

EGFP‐BMSCs were isolated from the bone marrow of SD rates (Rat Resource and Research Center). Specific‐pathogen‐free male Sprague–Dawley rats weighing 200–250 g were divided into three groups: (*i*) NS+PBS group: three rats were injected *via* the tail vein with PBS immediately after intratracheal NS instillation; (*ii*) NS+BMSC group: three rats were injected *via* the tail vein with GFP‐labelled BMSCs (5 × 10^6^/rat) after intratracheal NS instillation; and (*iii*) Injury+BMSC group: three rats were injected *via* the tail vein with GFP‐labelled BMSCs (5 × 10^6^/rat) immediately after intratracheal CASP instillation. The animals were anaesthetized by the intraperitoneal injection of 10% chloral hydrate (4 ml/kg) and killed by aortic bleeding. The lungs of the rats were harvested 4 and 12 hrs after the instillation of NS or CASP for frozen sectioning. The lung tissues were fixed with 4% paraformaldehyde in 0.1 M PBS, pH 7.4, for 4 hrs. The tissues were cryoprotected in 30% sucrose in 0.1 M PBS overnight and then embedded in an OCT compound (VWR International, Westchester, PA, USA) and cryosectioned into 5 μm sections on Superfrost slides (Fisher Scientific, MA, USA). The slides were stored at −80°C until ready to be viewed and then thawed at room temperature. The sections were rehydrated with PBS buffer. Fluorescent tissue was examined under a Nikon Eclipse TE 300 microscope (Nikon, Tokyo, Japan), and images were captured with a CCD camera.

### 15d‐PGJ_2_ treatment

Specific‐pathogen‐free male Sprague–Dawley rats weighing 200–250 g were divided into two groups: (*i*) Injury+PBS group: five rats were treated with PBS *via* an intraperitoneal injection immediately after intratracheal CASP instillation; and (*ii*) Injury+PBS group: five rats were treated with 15d‐PGJ_2_ (1 mg/kg) *via* an intraperitoneal injection immediately after intratracheal CASP instillation. The blood, bronchoalveolar lavage fluid (BALF) and lungs were harvested 4 and 12 hrs after CASP injury for histopathological analysis, arterial blood gas analysis, cell count and total protein measurement in BALF and cytokine measurement by ELISA.

### Blood gas analysis

The animals were anaesthetized by the intraperitoneal injection of 10% chloral hydrate (4 ml/kg), and arterial blood samples for the measurement of partial oxygen tension (PaO_2_) were taken from the abdominal aortic of rats and analysed with a GEM Premier 3000 blood gas analyser (International Lab, South San Francisco, CA, USA) 4 and 12 hrs after instillation of NS or CASP.

### Cell counts in lung and BALF

The animals were anaesthetized by the intraperitoneal injection of 10% chloral hydrate (4 ml/kg) 4 and 12 hrs after instillation of NS or CASP. After the arterial blood samples were collected, the trachea of the rats was exposed, and a 16 gauge cannula (BD Biosciences, Franklin lakes, NJ, USA) was inserted into the trachea and sutured in position above the carina. Then, the anaesthetized animals were killed by abdominal aortic bleeding, and blood samples were collected. The right lung was ligatured and excised. Two leaves of the right lung were stored in liquid nitrogen. One leaf of the right lung was used for a lung wet‐to‐dry ratio calculation, and the remaining leaf was immediately fixed in 10% neutral‐buffered formalin for histology and an immunohistochemistry assay. The whole left lung, which was used for BALF, was flushed back and forth with 2 ml of cold PBS three times. Lavage fluid from three flushes was pooled and centrifuged at 250 × g for 10 min. at 4°C. After the pooled BALF was centrifuged, the supernatants were harvested. The remaining cell pellets were resuspended in 0.5 ml of cold PBS. Then, 20 μl of the cell suspension was used to count the total number of cells using a haemocytometer. The remaining suspension was used for cell smears in slides by cytospin with a TXT3 cytospin machine (stxiangyilxj, Hunan, China) at 250 × g for 10 min., and then, the cells in the slides were stained using Wright‐Giemsa (Nanjing Jiancheng, Nanjing, China); the subpopulation of the white blood cells was counted from the total 200 cell count of a representative portion of the slides 15.

### Measurement of the lung wet‐to‐dry ratio

One leaf of the right lung, which was used for the wet‐to‐dry ratio calculation, was placed in a microtube, weighed, dried at 60°C for 72 hrs and weighed again. The wet lung was divided by the dry lung mass, representing the wet‐dry lung ratio and indicating the fraction of the wet lung weight owing to water.

### Histopathology

After fixation in 10% neutral‐buffered formalin, the lung was embedded in paraffin, cut into 5 μm sections, and stained with haematoxylin and eosin. An investigator who was unaware of the group assignments graded the level of lung injury using ATS guidelines to score the histological changes 17.

### ELISA

Blood sample was drawn from abdominal aorta, and the supernatants were collected after the blood was centrifuged at 800 × g for 15 min. at 4°C. The serum was stored at −80°C until use. Tumour necrosis factor (TNF)‐α, CINC‐1, IL‐10 and 15d‐PGJ_2_ were measured in both the BALF and serum samples with ELISA kits (TNF‐α and IL‐10 from ExCell Biology, Shanghai, China; CINC‐1 from R&D Systems, Santa monica, CA, USA; 15d‐PGJ_2_ from Enzo Life Sciences) following the manufacturer's instructions. Protein, as a marker of endothelial and epithelial permeability, was measured in the BALF with a Bradford protein assay kit (Bio‐Rad, Hercules, CA, USA).

### Immunohistochemistry

Lung section slides were deparaffinized and then exposed to 0.2% Triton X‐100 in PBS for 5 min. The slides were further blocked with 3% goat serum, 2% horse serum and 3% bovine serum albumin (BSA)/0.1% Tween 20 in 4× SSC for 20 min. A primary antibody to PPAR‐γ (Abcam, Cambridge, UK, in 2× SSC/1% BSA/0.1% Tween 20) was added for 1 hr at 37°C at a 1:100 dilution. The slides were washed three times with PBS and then blocked with 3% BSA/0.1% Tween 20 in 4× SSC for 20 min. A FITC‐conjugated secondary Ab (in 2× SSC/1% BSA/0.1% Tween 20) was added for 1 hr at 37°C. Control slides were stained with isotype Ab. The slides were washed three times with PBS and mounted on glass slides in FluoroGuard antifade reagent (Bio‐Rad), and coverslips were applied. Laser confocal fluorescence microscopy (OLYMPUS A, FV1000; Olympus, Tokyo, Japan) was performed (*n* = 3 per group per time‐point).

### Western blot

Lung tissues homogenates were prepared by pulverizing frozen tissue and placing it in a lysis buffer (20 mM 4‐(2‐Hydroxyethyl)‐1‐piperazineethanesulfonic acid (HEPES) (pH 7.4), 150 mM NaCl, 10% glycerol, 1% Triton X‐100, 1 mM ethylenebis tetraacetic acid (EGTA), and 1.5 mM MgCl_2_) containing a protease inhibitor mix (1 mM phenylmethanesulfonyl fluoride, 10 mM sodium pyrophosphate, 50 mM NaF, 2 mM sodium orthovanadate and 1 μM Lactacystin) at a concentration of 33 mg/ml. The samples were centrifuged at 800 × g for 10 min. at 4°C), and debris and nuclei were removed. The supernatants were removed and centrifuged at 17,000 × g for 20 min. at 4°C. The total protein content of the resulting supernatants was quantified using the method of bicinchoninic acid with a Beckman Coulter spectrophotometer. Then, the denatured proteins were loaded and run on a 4–12% gradient Bis‐Tris gel (Boston Biomedical, Inc., Boston, MA, USA). The gel was first run at 120 V for 15 min. and then changed to 160 V using an electrophoresis system (Bio‐Rad). The proteins were then transferred to a nitrocellulose membrane and incubated with blocking buffer for 1 hr. The membrane was then exposed to rabbit polyclonal primary antibodies for PPAR‐γ, p‐p65, TNF‐α and GAPDH (Abcam) at a 1/1000 dilution overnight at 4°C. The membrane was washed and then incubated with anti‐rabbit HRP‐labelled antibodies (GE Healthcare, Shanghai, China) at a 1/2500 dilution for 30 min. The protein bands were subsequently visualized with an ECL kit (Pierce).

### *In vitro* experiments on BMSCs

The BMSCs at passage 5 were randomized into control and injury groups. The injury groups were randomly allocated into three groups by different stimulation concentrations of TNF‐α (5, 10 or 20 ng/ml respectively). Then, the cells were divided into three groups by different observing times: 6, 12 or 24 hrs after stimulation. The level of 15d‐PGJ_2_ in the supernatant of the culture liquid was detected by ELISA with a 15d‐PGJ_2_ ELISA kit according to the manufacturer's protocol (Enzo Life Sciences).

### Statistical analysis

The data were presented as the means ± S.E.M. Statistical analysis was performed with the SPSS 19.0 software (International Business Machines Corporation, Armonk, NY, USA). For continuous variables with a normal distribution, data were analysed *via* one‐way anova and an unpaired *t*‐test or a Mann–Whitney test. Continuous variables that were not normally distributed were analysed using the Wilcoxon‐Rank test. *P* < 0.05 was considered statistically significant.

## Results

### Characterization of BMSCs

**Figure 1:**
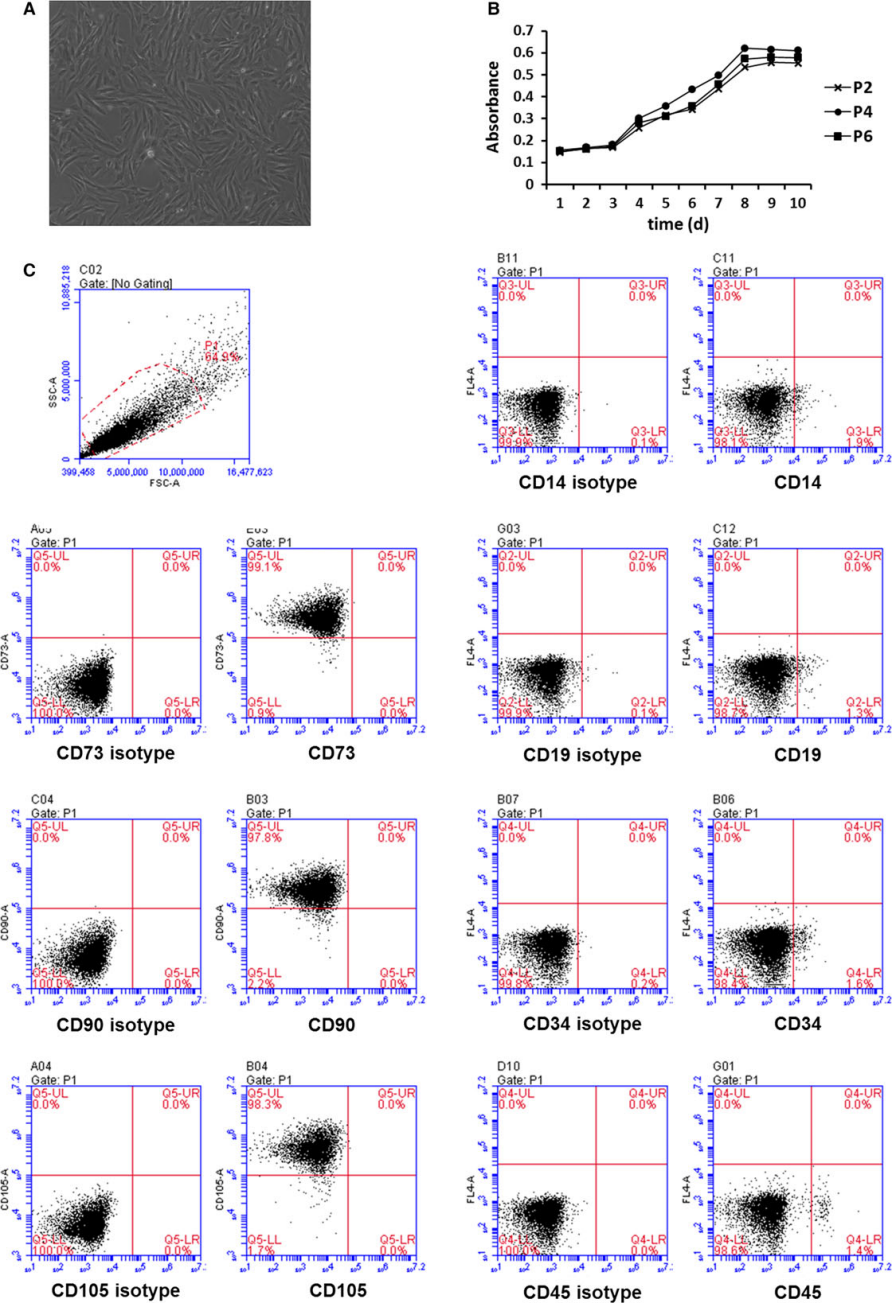
Characterization of BMSCs. BMSCs was isolated from Sprague–Dawley rats bone marrow. The morphology of BMSCs at passage 5 (100×) (**A**). The growth curves of BMSCs at passage 2, 4, 6 were determined by a CCK‐8 kit in accordance with the manufacturer's instructions (**B**). BMSCs were stained with CD14, CD19, CD34, CD45, CD73, CD90, CD105 antibodies and received FACS analysis (**C**).

The BMSCs that were cultured at passage 5 showed a homogeneous spindle‐shaped morphology (Fig. 1A). The BMSCs grew most rapidly 3–8 days after seeding, and the growth curve of the BMSCs resembled an s‐shaped curve (Fig. 1B). FACS analysis showed that the cells were uniformly negative for the haematopoietic markers CD14 (1.9%), CD19 (1.3%), CD34 (1.6%) and CD45 (1.4%) and positive for stem cell antigens CD73 (99.1%), CD90 (97.8%) and CD105 (98.3%) (Fig. 1C). Thus, the cell population that was used in this study represents a phenotype that is consistent with that of BMSCs.

### Mortality of rats

The mortality was 0% at 4 and 12 hrs after injury with NS instillation in the PBS treatment group. The mortality was 16.7% at 4 and 12 hrs after injury with CASP instillation in the PBS treatment group. The mortality was 0% at 4 hrs after injury and 16.7% at 12 hrs after injury with CASP instillation in the BMSC treatment group.

### Treatment with BMSCs reduces lung injury and inflammation

**Figure 2:**
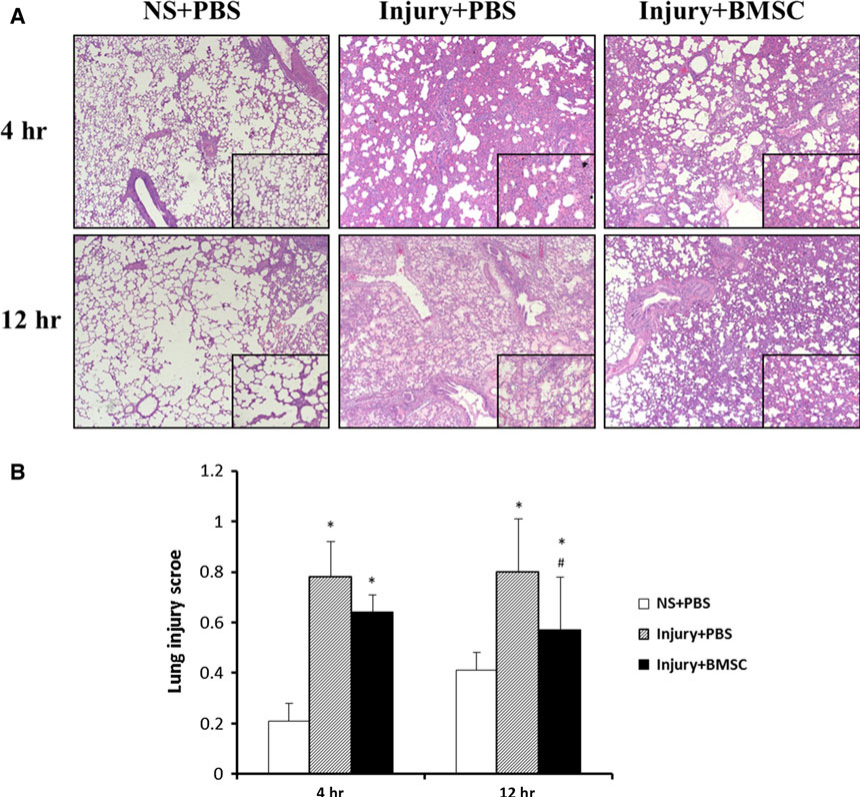
Treatment with BMSCs reduced lung injury and inflammation. The right lungs of rats were harvested 4 and 12 hrs after instillation of NS or CASP and inflation fixed for haematoxylin and eosin staining. Representative pictures were taken at a magnification of 50× and inserts at 200× (**A**). Qualitification analysis of lung injury scores (**B**). Data were presented as means ± S.E.M. (*n* = 6 rats/group). *P < 0.05 *versus* NS+PBS group; #*P* < 0.05 *versus* injury + PBS group.

Histological analysis showed that there were alveolar oedema, extensive consolidation and peri‐bronchial and peri‐vascular infiltration in lung tissues in CASP‐instillation rats compared to control NS instillation rats 4 and 12 hrs after instillation (Fig. 2A). Treatment with BMSCs significantly decreased the infiltration of inflammatory cells in lung tissues from CASP‐injured rats compared to those of the PBS treatment group (Fig. 2A). A quantification analysis showed that the inflammatory scores were significantly lower in the lung tissues from BMSC‐treated CASP‐injured rats than in those from PBS‐treated CASP‐injured rats (Fig. 2B).

### Treatment with BMSCs alleviates biochemical changes of lung in CASP‐injured rats

**Figure 3:**
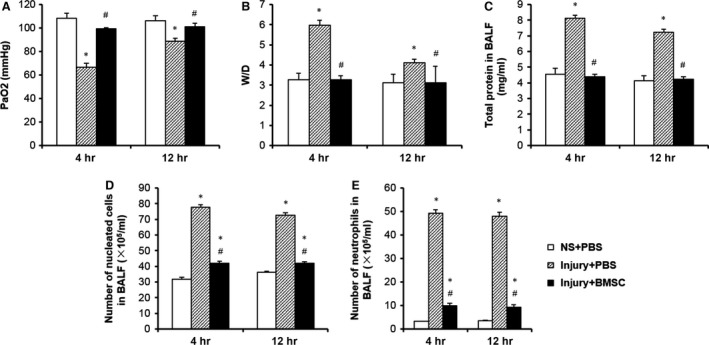
Treatment with BMSCs alleviated biochemical changes of lung in CASP‐injured rats. Blood gas analysis (**A**), lung wet‐to‐dry ratio (**B**), total protein concentration (**C**), nucleated cells count in BALF (**D**) and neutrophils count (**E**) in BALF were analysed 4 and 12 hrs after instillation of NS or CASP. Data were presented as mean ± S.E.M. (*n* = 6 rats/group). *P < 0.05 *versus* NS+PBS group; #*P* < 0.05 *versus* injury + PBS group.

A blood gas assay showed that CASP injection significantly decreased the PaO_2_ in the rats compared to NS injection and that treatment with BMSCs increased PaO_2_ in the CASP‐injured rats (Fig. 3A). Combined acid and small gastric particles injection significantly increased the wet/dry weight ratio (W/D) of the lungs, the level of the total proteins in BALF, and the number of nucleated cells and neutrophils 4 and 12 hrs after CASP injury, whereas the BMSC treatment significantly decreased the W/D of the lungs, the level of total proteins in BALF and the number of nucleated cells and neutrophils 4 and 12 hrs after CASP injury (Fig. 3B–E). The results showed that BMSC treatment alleviates biochemical changes in the lung in CASP injury rats.

### Administration of BMSCs reduces the systemic and local inflammatory response of CASP injury

**Figure 4:**
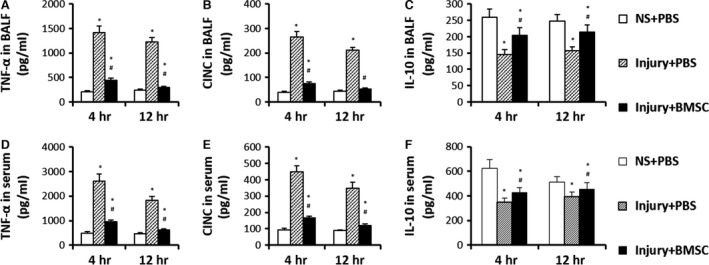
Administration of BMSCs reduced the systemic and local inflammatory response of CASP injury. TNF‐α (**A** and **D**), CINC (**B** and **E**), IL‐10 (**C** and **F**) in BALF and serum were analysed 4 and 12 hrs after instillation of NS or CASP. Data were presented as means ± S.E.M. (*n* = 6 rats/group). *P < 0.05 *versus* NS+PBS group; #*P* < 0.05 *versus* injury + PBS group.

The levels of the pro‐inflammatory cytokines TNF‐α and CINC‐1 in BALF and serum 4 and 12 hrs after CASP injury were significantly higher in the injury + PBS group than in the NS + PBS group. The levels of the pro‐inflammatory cytokines TNF‐α and CINC‐1 in BALF and serum 4 and 12 hrs after CASP injury were significantly lower in the injury + BMSC group than in the injury+PBS group (Fig. 4A, B, D and E). The anti‐inflammatory cytokine IL‐10 in BALF and serum 4 and 12 hrs after CASP injury were significantly higher in the injury + BMSC group than in the injury+PBS group (Fig. 4C and F). These results suggest that treatment with BMSCs inhibits the production of pro‐inflammatory cytokines and increases the production of anti‐inflammatory cytokines in CASP injury.

### BMSCs reduce lung injury and inflammation *via* regulating inflammatory cytokines

**Figure 5:**
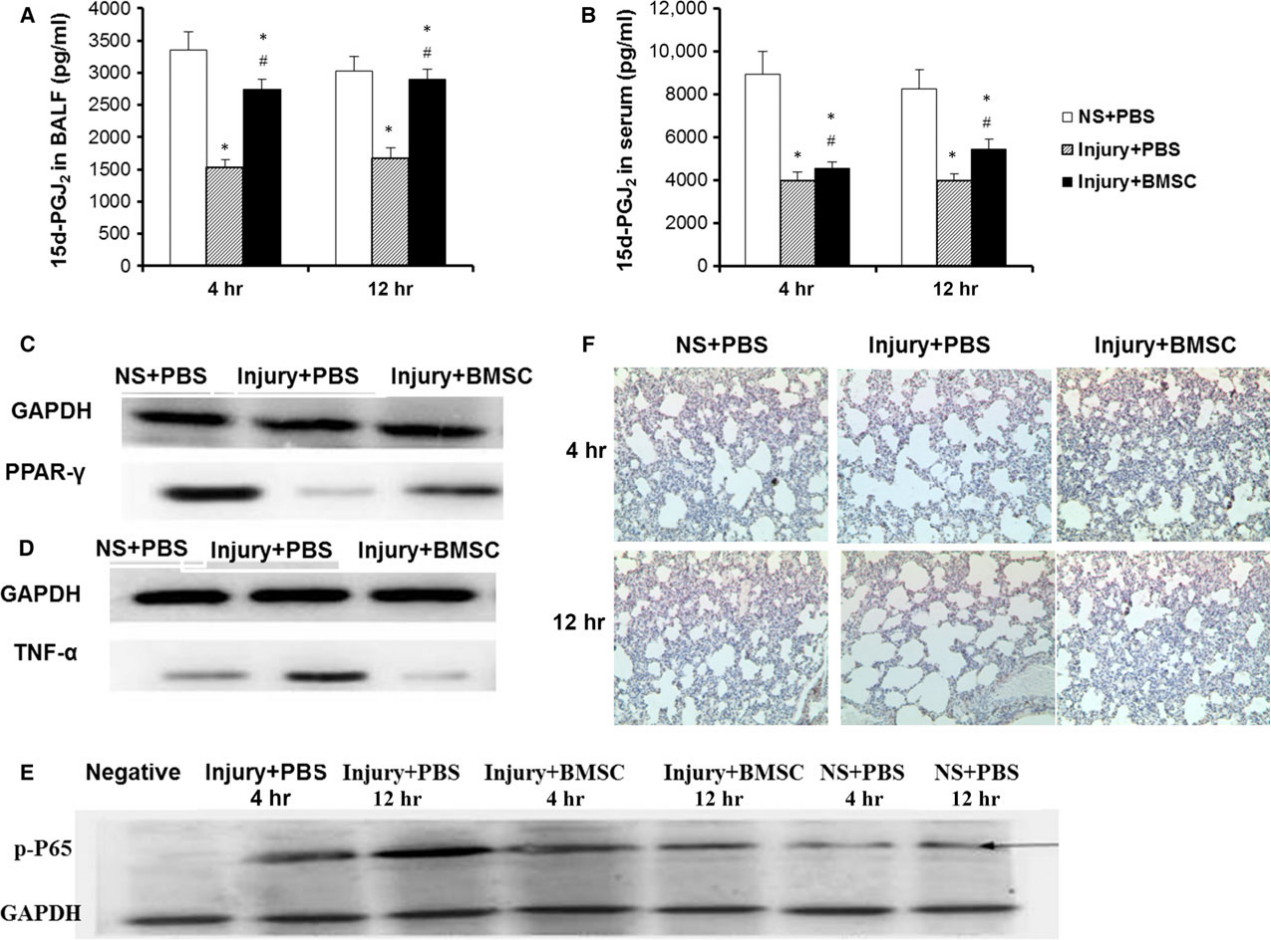
BMSCs reduced lung injury and inflammation *via* regulating inflammatory cytokines. The 15d‐PGJ2 levels in BALF (**A**) and serum (**B**) were detected 4 and 12 hrs after instillation of NS or CASP. The protein expression of PPAR‐γ (**C**), TNF‐α (**D**) and NF‐κB p65 (**E**) in lung tissues were determined by Western blot. The expression of PPAR‐γ in lung tissues was analysed by immunohistochemistry (magnification 400×) (**F**). Data were presented as means ± S.E.M. (*n* = 6 rats/group). *P < 0.05 *versus* NS+PBS group; #*P* < 0.05 *versus* injury + PBS group.

ELISA showed that the levels of the prostaglandin 15d‐PGJ_2_ in BALF and serum decreased at 4 and 12 hrs in the rats with CASP injury and that the levels of 15d‐PGJ_2_ recovered significantly in the rats with BMSC treatment (Fig. 5A and B). Western blot showed that the expression of PPAR‐γ in lung tissues decreased at 4 hrs in rats with CASP injury and was recovered in rats that received BMSC treatment (Fig. 5C). The level of the TNF‐α protein increased in rats with CASP injury and decreased in rats that received BMSC treatment (Fig. 5D). NF‐κB p65 expression in the lung tissues increased at 4 and 12 hrs in the rats suffering from CASP injury compared to rats that received a NS injury. Bone‐marrow‐derived mesenchymal stem cell treatment significantly decreased the NF‐κB p65 levels compared to the control treatment (Fig. 5E). The immunohistochemical staining of the lung tissue showed that the expression of PPAR‐γ decreased 4 and 12 hrs after CASP injury and increased in CASP injury with BMSC treatment compared to CASP injury alone (Fig. 5F).

### The levels of 15d‐PGJ_2_ that were secreted by BMSCs with TNF‐α stimulation *in vitro*

**Figure 6:**
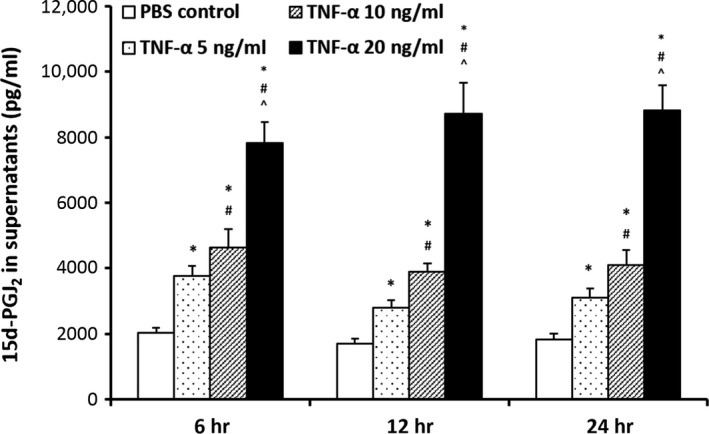
The levels of 15d‐PGJ2 that were secreted by BMSCs with TNF‐α stimulation *in vitro*. BMSCs (5 × 105/well) were stimulated with different concentrations of TNF‐α in six‐well plate with non‐serum DMEM medium. The 15d‐PGJ2 levels of supernatant were determined by ELISA after 24 hrs stimulation with TNF‐α. Data were presented as means ± S.E.M. *P < 0.05 *versus* PBS control group; #*P* < 0.05 *versus* TNF‐α 5 ng/ml group; ^*P* < 0.05 *versus* TNF‐α 10 ng/ml group.

To further investigate the mechanism by which BMSCs inhibited inflammation, we measured the levels of 15d‐PGJ_2_ in the supernatants of BMSCs that were stimulated with TNF‐α. Tumour necrosis factor‐α stimulation significantly increased the levels of 15d‐PGJ_2_ in the supernatants of BMSCs in a dose‐dependent manner, but not a time‐dependent manner (Fig. 6). These results suggest that BMSCs exhibit anti‐inflammatory activity by secreting 15d‐PGJ_2_ under inflammatory conditions (TNF‐α).

### BMSCs migrate to inflammatory lung tissues

**Figure 7:**
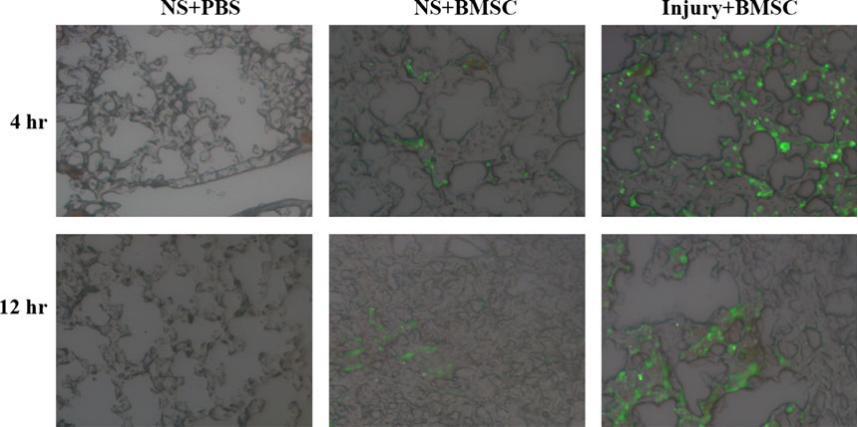
BMSCs migrated to inflammatory lung tissues. Rats were injected *via* the tail vein with GFP+ BMSCs (5 × 106/rat) or PBS immediately after being instilled with NS or CASP intratracheally (*n* = 3 rats/group). The lungs of rats were harvested for frozen sections 4 and 12 hrs after BMSCs administration. Fluorescent tissue was examined using a Nikon Eclipse TE 300 microscope, and images were captured with a CCD camera and Bioquant Software. The experiment was repeated three times. Representative pictures were taken at a magnification of 400×.

To further determine the systemic or local effect of BMSCs on lung inflammation, we injected EGFP^+^ BMSCs into Sprague–Dawley rats that were instilled with CASP by the tail vein. A large number of EGFP^+^ BMSCs were observed in the lung tissues of rats in both the control NS‐ and CASP‐injected groups but not in the non‐EGFP‐BMSC‐injected group (Fig. 7). More EGFP^+^ BMSCs were detected in the lung tissues of CASP‐injured rats than in those of the NS control rats 4 and 12 hrs after the injection of EGFP^−^ BMSCs (Fig. 7). These results demonstrate that BMSCs can migrate to and remain in the inflammatory lung tissues in our model.

### The administration of 15d‐PGJ_2_ reduces the systemic and local inflammatory response of CASP injury

**Figure 8:**
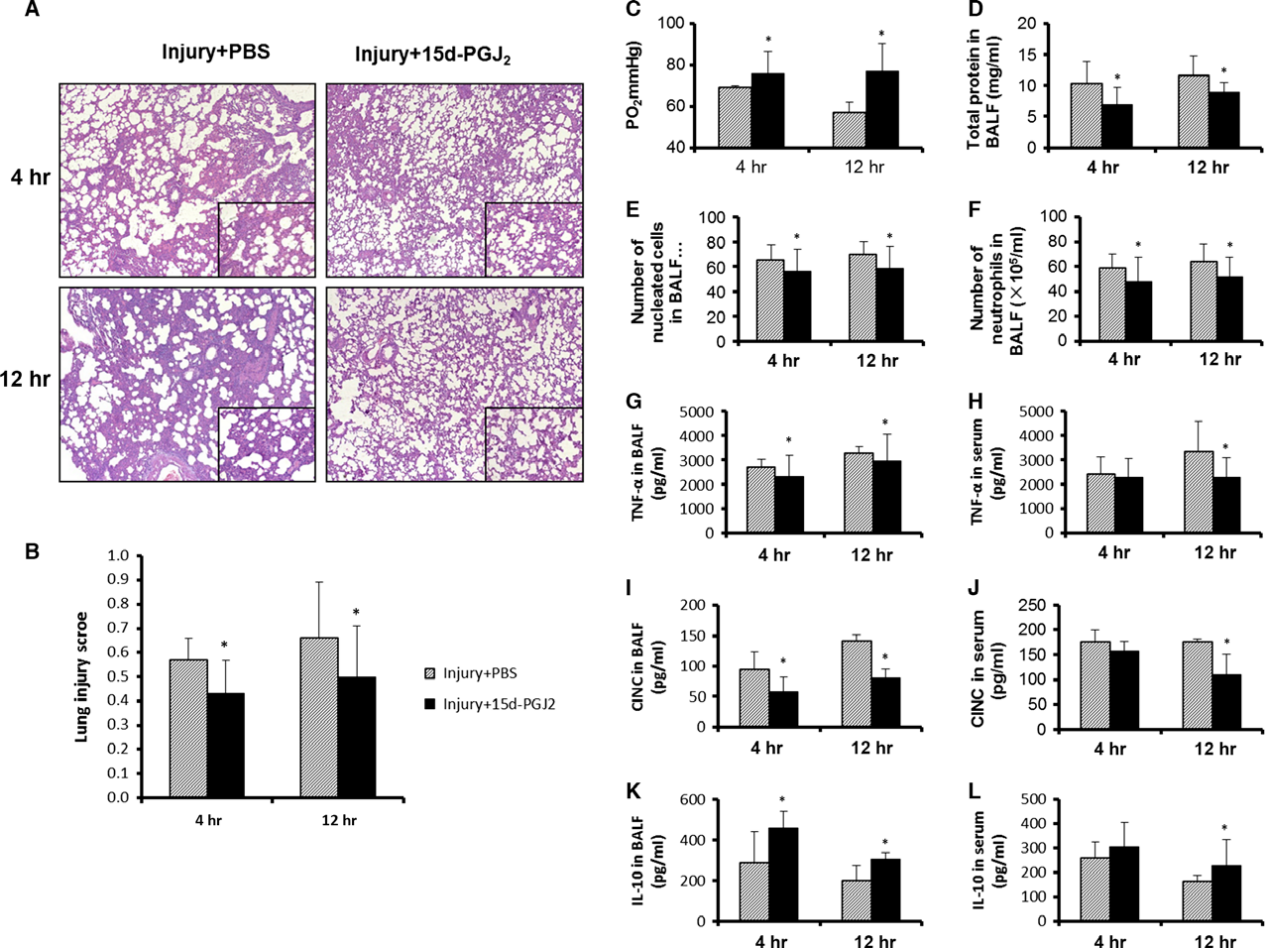
The administration of 15d‐PGJ2 reduced the systemic and local inflammatory response of CASP injury. Rats were treated with 15d‐PGJ2 (1 mg/kg) or PBS *via* an intraperitoneal injection immediately after intratracheal CASP instillation. The blood, BALF and lungs were harvested 4 and 12 hrs after CASP injury. Representative pictures were taken at a magnification of 50× and inserts at 200× (**A**). Lung injury scores were determined by blinded evaluation (**B**). Blood gas analysis (**C**), total protein concentration (**D**), nucleated cells count (**E**) and neutrophils count (**F**) in BALF were analysed. The levels of TNF‐α, CINC and IL‐10 in BALF and serum were measured by ELISA (**G**–**L**). Data were presented as means ± S.E.M. (*n* = 5 rats/group). *P < 0.05 *versus* injury + PBS group.

To further investigate whether BMSC‐derived 15d‐PGJ_2_ plays an important role in the inhibition of lung inflammation, we peritoneally injected 15d‐PGJ_2_ into Sprague–Dawley rats. Treatment with 15d‐PGJ_2_ significantly alleviated acute lung jury and lung inflammation compared to the control treatment (Fig. 8A and B). Treatment with 15d‐PGJ_2_ significantly increased the level of PaO_2_ (Fig. 8C) in serum and reduced the level of total protein (Fig. 8D) as well as the number of nucleated cells (Fig. 8E) and neutrophils (Fig. 8F) in the BALF of the CASP‐injured rats. Furthermore, the 15d‐PGJ_2_ treatment significantly decreased the levels of TNF‐α and CINC‐1 and increased the level of IL‐10 in BALF and serum 4 and 12 hrs after CASP injury (Fig. 8G–L). These results show that the 15d‐PGJ_2_ treatment alleviates systemic and local inflammation in CASP‐injured rats.

## Discussion

Many recent studies have reported the effects of BMSCs on ALI 18, 19. However, few studies have investigated the effects of BMSCs on aspiration lung injury 20. It remains unknown how BMSCs protect against ALI. One potential mechanism is that the capability of BMSCs to regenerate plays an important role in ALI. It is believed that BMSCs can migrate to the injured lung tissue and then differentiate into functional cells, which replace damaged lung cells in the injured tissues. However, it is still unclear whether the number of BMSCs that differentiate into functional cells in damaged tissues is enough to repair the injured tissues and whether the differentiated cells have normal functions in lung tissue cells. Furthermore, because it will take some time for BMSCs to complete regeneration and repair, the time that is required for BMSC activity may not coincide with the occurrence of ALI. Therefore, it is possible that the protective effect of BMSCs on ALI is because of the anti‐inflammatory and immune regulation function of BMSCs, especially in the early stages of ALI 13. There are few studies that have investigated whether the anti‐inflammatory effect of BMSCs plays a main role in ALI.

The nature of gastric particulate‐induced aspiration lung injury is an acute inflammatory response. This injury has its unique characteristics, including a two‐phase injury curve that corresponds to an initial chemical acid burn, followed by a secondary injury that is mediated by neutrophils, other inflammatory cells and a variety of inflammatory factors 4, 7. Our results showed that the W/D of the lungs, the level of total proteins, the number of total cells and the neutrophil counts in BALF increased in rats with intratracheal CASP instillation compared to those in rats that received the NS control. The PaO_2_ decreased in the rats that were instilled with the CASP compared to that in the rats that received the NS control. The lung pathological examination showed that alveolar oedema, exudation and lung inflammatory infiltration were evident and that the pathological lung injury score was higher in the CASP group than in the NS control group. The levels of the pro‐inflammatory cytokines TNF‐α and CINC‐1 in BALF and plasma were also significantly higher in the CASP‐injured rats than those in the rats that received the NS control. The data demonstrated that CASP‐induced aspiration lung injury model rats were successfully established. Bone‐marrow‐derived mesenchymal stem cell transplantation significantly increased PaO_2_ and significantly decreased the W/D of the lung, the level of total proteins, the total cell counts and the neutrophil counts in BALF in CASP‐induced aspiration rats. The lung pathological analysis showed that alveolar oedema, exudation and lung inflammatory infiltration were significantly reduced in lung tissues from BMSC‐transplanted rats. The pathological lung injury scores significantly decreased in rats that were treated with BMSCs compared to those in rats that were treated with PBS. These results demonstrate that BMSCs can inhibit CASP‐induced lung inflammation.

Although a large number of studies have reported that multiple mechanisms are involved in the anti‐inflammatory, anti‐oxidative stress and immuno‐modulatory functions of BMSCs, the exact mechanisms are not fully understood. Some studies have reported that the expression of COX‐2 increased under the stimulation of some inflammatory factors, increasing the release of PGE_2_ 14, 15. PGE_2_ then induced macrophages to release anti‐inflammatory IL‐10, which plays a protective role. PGE_2_ is constitutively expressed in cultured BMSCs 21. In addition to PGE_2_, it is possible that other PG compounds from BMSCs may play roles in their anti‐inflammatory and immuno‐modulatory functions. 15d‐PGJ_2_, which is a prostaglandin compound that is generated by the spontaneous dehydration of PGD_2_, has an important protective effect on inflammation and oxidative stress damage 9, 10, 11. We found that CASP injury decreases the 15d‐PGJ_2_ levels, whereas BMSCs treatment restores the 15d‐PGJ_2_ levels, suggesting that BMSC‐derived 15d‐PGJ_2_ may play an important role in the inhibition of lung inflammation. The protective role of 15d‐PGJ_2_ in lung injury was further confirmed through an experiment in which 15d‐PGJ_2_ was directly injected into CASP‐injured rats. 15d‐PGJ_2_ treatment significantly reduced CASP‐induced lung inflammation and the production of pro‐inflammatory cytokines, confirming that 15d‐PGJ_2_ plays a major role in the BMSC‐mediated inhibition of lung inflammation.

In this study, we also observed an interesting phenomenon in which BMSC treatment increased the expression of PPAR‐γ in CASP‐injured rats. PPAR‐γ is a nuclear receptor that has pleiotropic effects on lipid metabolism, inflammation and cell proliferation. 15d‐PGJ_2_ is an endogenous PPARγ ligand. 15d‐PGJ_2_ activates PPAR‐γ, resulting in the inhibition of the NF‐κB and AP‐1 transcription factors, exhibiting anti‐inflammatory effects. NF‐κB is a key transcription factor that mediates pro‐inflammatory cytokine production 22. Our results showed that NF‐kB p65 expression in lung tissues significantly increased in the CASP‐aspirated rats and that BMSC transplantation significantly decreased NF‐kB p65 expression and cytokine TNF‐α production. The increased levels of PPARγ and 15d‐PGJ_2_ induced by BMSCs may amplify the protective effect of BMSCs in lung inflammation by increasing PPARγ and 15d‐PGJ_2_ binding.

We also noted a baseline level of 15d‐PGJ_2_ in lung tissues. The cyclopentenone prostaglandin 15d‐PGJ_2_ is the end‐product metabolite of prostaglandin D_2_ (PGD_2_) and is produced by a variety of cells, including mast cells, T cells, alveolar macrophages, endothelial cells and epithelial cells. A recent study 23 showed that PGD_2_, the source of 15d‐PGJ_2_, may derive from alveolar non‐haematopoietic lineage cells (i.e. endothelial cells and epithelial cells) and promotes the vascular barrier function of lung tissues during the early‐phase (day 1), whereas neutrophil‐derived PGD_2_ attenuates its own infiltration and cytokine expression during the later phase (day 3). Treatment with either an agonist to the PGD_2_ receptor, DP, or a degradation product of PGD_2_, 15d‐PGJ_2_, exerted a therapeutic action against ALI. It is likely that the baseline levels of 15d‐PGJ_2_ come from the PGD_2_ of endothelial and epithelial cells in the lung tissues. Baseline 15d‐PGJ_2_ may have physiological effects that protect lung tissues from pathological injury. A possible explanation based on the results of our study is that CASP injury damages the endothelial and epithelial cells of lung tissues, decreasing the 15d‐PGJ_2_ levels. The transplanted BMSCs that migrate to local lung tissues could be activated by local cytokines (such as TNF‐α) and secreted 15d‐PGJ_2_, resulting in the restoration of the 15d‐PGJ_2_ levels. In fact, TNF‐α stimulated BMSCs to produce 15d‐PGJ_2_ in a dose‐dependent manner *in vitro*. *In vivo*, treatment with BMSCs increased the levels of 15d‐PGJ_2_, suggesting that BMSCs are a major source of 15d‐PGJ_2_. However, we did not observe a negative relationship between TNF‐α and 15d‐PGJ_2_ in BMSC‐treated rats, indicating that other factors may also contribute to BMSC stimulation for 15d‐PGJ_2_ production. Therefore, further investigation of the cell origin of 15d‐PGJ_2_ and exact mechanisms of BMSCs is required.

In summary, our study shows that BMSC engraftment inhibits CASP‐induced aspiration lung injury. BMSC‐derived 15d‐PGJ_2_ plays an important role in the inhibition of lung inflammation by activating the PPAR‐γ receptor, reducing the production of proinflammatory cytokines. Although we demonstrated the protective effects of BMSCs in a CASP‐induced aspiration lung injury model, it is still not clear whether BMSCs maintain their phenotype or differentiate after 4 and 12 hrs of stimulation. Second, we do not know the source of the baseline 15d‐PGJ_2_ or how the CASP challenge decreased the level of 15d‐PGJ_2_ in this model. Finally, there is no justification from our study for the clinical application of BMSC treatment immediately after intratracheal CASP injury.

## Conflict of interest

The authors confirm no conflicts of interest.
